# Utilization of base deficit and reliability of base deficit as a surrogate for serum lactate in the peri-operative setting

**DOI:** 10.1186/1471-2253-10-16

**Published:** 2010-09-09

**Authors:** Lakhmir S Chawla, Amirali Nader, Todd Nelson, Trusha Govindji, Ryan Wilson, Sonia Szlyk, Aline Nguyen, Christopher Junker, Michael G Seneff

**Affiliations:** 1 Department of Critical Care Medicine and Anesthesiology George Washington University Medical Center, Washington D.C., USA; 2 Division of Renal Diseases and Hypertension, Department of Medicine George Washington University Medical Center, Washington D.C., USA

## Abstract

**Background:**

Base deficit (BD) is commonly used in the operating room (OR) as an endpoint of resuscitation. BD is used as a surrogate marker for the accumulation of lactic acid(Lac). However, the BD can be affected by large amounts of saline.

**Methods:**

We conducted a survey of anesthesiologists regarding the use of BD. We also studied the reliability of BD to determine the presence of hyperlactatemia (HL). Patients undergoing general anesthesia were eligible for enrollment if they were receiving an arterial line as part of their routine care. If an arterial blood gas was drawn by the operative team as part of the routine care, the remainder of the unused blood was also used to measure Lac.

**Results:**

*Survey*: 73 staff anesthesiologists were surveyed. Over 70% of respondents used BD as an endpoint of resuscitation.

*Base Deficit Study*: 35 patients were enrolled resulting in 88 arterial blood gases with corresponding Lac. Mean age was 61.4 ± 14.3 years, 43% were male. Mean pH was 7.39 ± 0.05, the mean bicarbonate was 23.0 ± 2.3 meq/L, the mean BD 1.34 ± 2.3, and the mean Lac was 1.58 ± 0.71 mmol/L. Mean ASA risk score was 3.16 ± 0.71. ROC area under the curve for base deficit to detect HL was 0.58.

**Conclusion:**

BD can often mislead the clinician as to the actual Lac. Lac can now be measured in the OR in real time. Therefore, if clinicians in the operative setting want to know the Lac, it should be measured directly.

## Background

Base deficit is the amount of base (in mmol) required to titrate a liter of whole arterial blood to a pH of 7.40. Base deficit as a measure of metabolic acidosis was first proposed by Anderson and Engel in 1960[1]. Over time, this assessment of metabolic acidosis has been incorporated into the standard information that is reported on a routine blood gas analysis. Base deficit has been advocated as a marker of resuscitation adequacy[2-6]. These recommendations are based on animal and human data that show that the base deficit is correlated with severity of injury and degree of hemorrhage[2,5-8]. As a consequence, base deficit is commonly used in the operating room (OR) as an endpoint of resuscitation. Because base deficit accounts for the buffering effects of hemoglobin and CO2, both of which may fluctuate significantly in long complex operative procedures, base deficit is used by anesthesiologists as a surrogate marker for metabolic acidosis. The rationale for base deficit as a marker of severity of illness is based on the fact that as patients suffer from hemorrhage and hypotension, oxygen delivery becomes inadequate forcing the onset of anaerobic metabolism. Anaerobic metabolism results in accumulation of lactic acid, and base deficit has been shown to correlate with serum lactate in models of shock[9]. However, the base deficit can be affected if large amounts of saline are administered which is common in peri-operative patients, and the base deficit does not always correlate with the serum lactate in trauma patients and patients who are critically ill[10-12]. In addition, base deficit as a surrogate for serum lactate can be confounded by renal dysfunction and ketoacidosis. We have previously studied the accuracy of base deficit as a surrogate for lactate in patients who are critically ill. However, we were unable to find any studies involving intra-operative patients that tested whether this relationship is maintained during surgery. Furthermore, the commonly held belief that the absence of base deficit rules out the presence of hyperlactatemia persists despite previous studies showing that base deficit is not effective at discriminating the presence or absence of hyperlactatemia[13-17]. Formal quantitative studies on frequency of use of base deficit in the OR and thresholds have not been conducted. We sought to better understand the utilization of base deficit as a clinical tool in the intra-operative setting.

## Methods

### Utilization Survey

**Figure 1:**
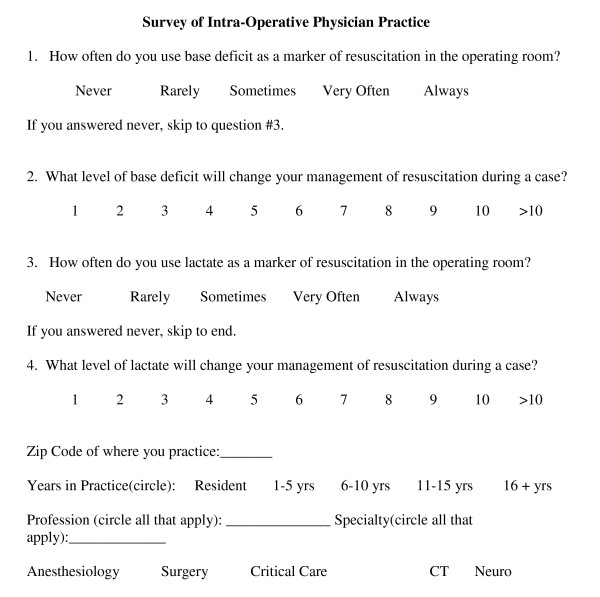
**Survey**.

We conducted a survey (Figure 1) of anesthesiology attendings and residents at 3 academic medical centers (Washington Hospital Center Hospital, Washington DC, George Washington University Medical Center, Washington DC, and University of North Carolina, Chapel Hill, NC). The survey was approved by the George Washington University Medical Center Institutional Review Board and granted a waiver of HIPAA (Health Insurance Portability and Accountability Act) and informed consent. No personal identifiable material was collected.

### Base Deficit Study

This part of the study was conducted from September 2007 to August 2008 in the GW hospital operating rooms. The study was approved by the Institutional Review Board (IRB) of the George Washington University Medical Center and the privacy officer of the hospital.

### Patients

Those patients undergoing general anesthesia were eligible for enrollment if they were receiving an arterial line as part of their routine care. Demographic, clinical, and operative information were collected.

### Study Procedures

If an arterial blood gas was drawn by the operative team as part of the routine care, the remainder of the unused blood was also used to measure serum lactate. Blood was immediately placed on ice and evaluated for pH, partial pressure or carbon dioxide/oxygen, oxygen saturation and lactate levels using standard blood gas analysis (ABL 700; Radiometer Americ Inc; Westlake, OH). All costs for this extra analysis were paid for the Department of Anesthesiology and Critical Care Medicine.

### Definitions and Analysis

For each patient, arterial serum lactate (Lac) was measured, and the standard base deficit (BD) was calculated from the arterial blood gas (ABL 700; Radiometer Americ Inc; Westlake, OH). Standard base deficit (BD) was determined using the modified Van Slyke equation.^1^

### Statistics

Proportions of patients with certain characteristics were compared using the chi-square test. We assessed the distribution of variables. Variables with normal distribution were compared using two-tailed unpaired t-tests, while data that did not conform to a normal distribution were compared using the Mann-Whitney rank sum test. BD and Lac were compared using Pearson correlations. Receiver operating characteristic (ROC) curves were determined for BD to detect the presence of hyperlactatemia. Hyperlactatemia was defined as a serum lactate greater than 2.0 mmol/L. Unless otherwise specified, all means are reported as ± S.D. All statistics were performed with SPSS 11.0 (SPSS, Chicago, IL.).

## Results

### Survey

**Figure 2:**
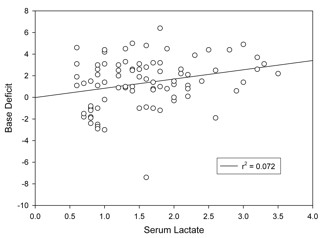
**Base Deficit v. Lactate**.

73 staff anesthesiologists and anesthesiologists in training were surveyed; 14 (19.2%) from Washington Hospital Center, 24 (32.9%) from the George Washington University Medical Center, and 35 (47.9%) from the University of North Carolina. Of the anesthesiologists surveyed, 37 (50.7%) were still in training, 7(9.6%) had 1-5 years of experience, 6 (8.2%) had 6-10 years of experience, 8(11%) had 11-15 years of experience, and 15(20.5%) had greater than 15 years of experience. Response to Questions 1, 2, 3, and 4 are outlined in Table 1, 2, 3, 4, and Figure 2.

**Table 1:** How frequently do you use base deficit as a marker of resuscitation in the Operating Room?

	N	**Percent**x
Never	11	15.1
Rarely	8	11.0
Sometimes	24	32.9
Very Often	26	35.6
Always	4	5.5

**Table 2:** What level of base deficit will change your management of resuscitation?

**N**	**Mean**	**SD**	**Inter-quartile Range**			
			
			**Q1**	**Q2**	**Q3**	**Q4**
62	6.31	2.53	0-5	5-6	6-8	8- >10

**Table 3:** How frequently do you use serum lactate as a marker of resuscitation in the Operating Room?

	N	Percent
Never	13	17.8
Rarely	16	21.9
Sometimes	26	35.6
Very Often	14	19.2
Always	4	5.5

**Table 4:** What level of serum lactate will change your management of resuscitation?

**N**	**Mean**	**SD**	**Inter-quartile Range**			
			
			**Q1**	**Q2**	**Q3**	**Q4**
60	4.10	2.11	0-3	3-4	4-5	5- >10

### Base Deficit Study

**Figure 3:**
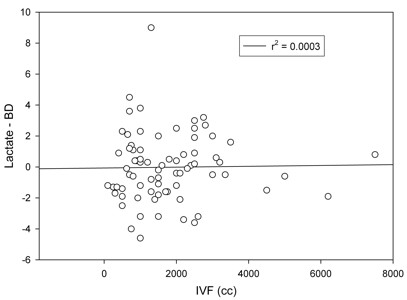
**Base Deficit-Lactate Difference v. Intravenous Fluids**.

**Figure 4:**
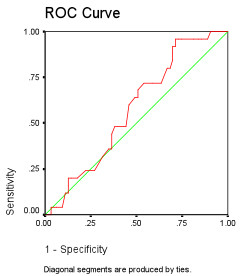
**ROC Area Under the Curve = 0.58**.

35 patients were consented and enrolled into the study. A total of 88 arterial blood gases were drawn with corresponding arterial serum lactate assessed as well. The mean age was 61.4 ± 14.3 years, 43% of the subjects were male. Of the patients, 19 (54.3%) were white, 10 (28.6%) were black, and 6 (17.1%) were from another ethnicity. The types of procedures were varied as shown in Table 5. The mean pH was 7.39 ± 0.05, the mean bicarbonate was 23.0 ± 2.3 meq/L, the mean standard base deficit was 1.34 ± 2.3, and the mean serum lactate was 1.58 ± 0.71 mmol/L. Of the 35 patients, six had and ASA risk score was 2 (n = 6), 3(n = 16), and 4(n = 11). For two subjects, no ASA risk score was recorded. As expected, base deficit correlated with pH and serum lactate (r = 0.40, p < 0.001, r = 0.27, p = 0.01, respectively) (Table 6). The correlation of BD versus serum lactate is shown in Figure 2. Figure 3 shows the difference of lactate and BD plotted against the amount of intravenous fluids given at the time of the measurement. The ROC area under the curve for base deficit to detect hyperlactatemia was 0.58 (Figure 4).

**Table 5:** Patient Characteristics

Study Population	
Age(mean,sd)	61.4 ± 14.3 years
Male	43%
Ethnicity (n,%)	
White	19 (54.3%)
Black	10 (28.6%)
Other	6 (17.1%)
Hemoglobin (mean,sd)	12.8 ± 1.7 g/dL
Serum Bicarbonate (mean,sd)	23.0 ± 2.3 meg/L
Albumin (mean,sd)	3.6 ± 1.5 g/dL
pH	7.39 ± 0.05
Base Deficit	1.34 ± 2.3
Serum Lactate (mmol/L)	1.58 ± 0.71
Hyperlactatemia n (%)	14 (40%)
ASA Class n(%)	
Class 2	6 (17.1%)
Class 3	16 (45.7%)
Class 4	11 (31.4%)
	
Operation n (%)	14 (40.0%)
CABG	2 (5.7%)
Other Cardiac Surgery	2 (5.7%)
Vascular Surgery	3 (8.5%)
Other	3 (8.5%) 11 (31.4%)

Legend: CABG(Coronary Artery Bypass Grafting), ASA Class (American Anesthesiology Association Risk Class)

**Table 6:** Correlations of Covariates

	BICARB	pH	BD	LAC
BICARB		0.10	-0.94**	0.26*
				
pH	0.10		-0.40**	0.16
				
BD	-0.94**	-0.40**		0.27*
				
LAC	0.26*	0.16	0.27*	

* p = 0.01

** p = 0.0001

Pearson Correlation is significant at the 0.01 level (2-tailed).

## Discussion

In this study, we showed that BD is used commonly by anesthesiologists as an endpoint of resuscitation ( > 70% of respondents use BD at least "sometimes"). The absolute value of BD used by different clinicians for assessing was variable, and many clinicians indicated that the absolute BD value was not as important as the trend. In comparison, fewer of these same respondents used serum lactate as an endpoint of resuscitation. In this study, we have demonstrated that BD is not a suitable surrogate for hyperlactatemia. These data are consistent with our previous findings in critically ill patients[11,12]. Moreover, our data indicate that the base deficit would often mislead the clinician as to the true serum lactate. We showed that the ROC area under the curve for BD to predict hyperlactatemia was 0.58 (0.50 is a coin toss); thus demonstrating the poor performance of BD as a surrogate for serum lactate (Figure 4).

These results are not surprising given previous literature assessing the capacity of base deficit as a surrogate for resuscitation and serum lactate in the critical care and trauma setting. We have previously shown that BD is not reliable as a surrogate for serum lactate in critically ill patients[11,12]. In addition BD has also been shown to be insensitive for detecting hyperlactatemia in surgical patients[18]. In a study by Mikulaschek et al of trauma patients, resuscitation decisions would have been wrong 33-58% of the time if BD or anion gap had been used as the sole criterion rather than serum lactate concentration[16]. Waters et al showed that in a study of surgical patients, elevations in BD without concomitant rise in lactate were attributed to hyperchloremia and were a manifestation of successful resuscitation rather than fluid deficit[19]. Base deficit is useful as a surrogate for resuscitation so long as it is a result of hyperlactatemia, but if it is caused by a hyperchloremic acidosis due to saline loading this relationship becomes discongrous[10]. In addition, base deficit as a surrogate for serum lactate can be confounded by renal dysfunction and ketoacidosis.

This study is the first to test this concept on patients undergoing general anesthesia in the operating room. These data are noteworthy because based on our survey results, clinicians routinely use BD as an endpoint of resuscitation. The assumptions underlying this physiology may be faulty in many patients because BD does not discriminate the cause of the acidosis. Since previous studies have shown that the accumulation of chloride from normal saline tends to increase the base deficit[10], many patients in the peri-operative setting may receive more volume and blood product transfusions than they should because the BD is increasing due to the saline loading, and the clinician may incorrectly interpret this rising BD as an indication that the patient in under resuscitated. In this study, we were unable to show that the discrepancy between BD and serum lactate worsened with the amount of volume resuscitation or the duration of the case (Figure 3). This is not surprising given the multitude of intra-operative activities that can affect acid base status ( e.g. resuscitation, saline from iv fluids, citrate from blood products, etc) However, since the level of agreement between BD and serum lactate was so poor at baseline, the effect would have had to be dramatic in order to detect it.

## Limitations

The study was limited by its modest size. Larger studies involving a wide variety of operative patients need to be conducted to validate these findings.

## Conclusions

In aggregate, the use of BD as a surrogate for serum lactate is not warranted. BD can often mislead the clinician as to the actual serum lactate concentration. With the widespread availability of point of service testing for lactate, the serum lactate can now be measured in the operating room in real time. Therefore, if clinicians in the operative setting want to know the serum lactate, it should be measured directly.

## Competing interests

The authors declare that they have no competing interests.

## Authors' contributions

AN, TD, TG, SS, RW, and AN collected and measured the data. CJ and MGS participated in the study design and writing of the manuscript. LSC conceived of the study, performed the statistical analysis, and participated in the writing of the manuscript. All authors read and approved the final manuscript

## Pre-publication history

The pre-publication history for this paper can be accessed here:

http://www.biomedcentral.com/1471-2253/10/16/prepub
